# Senior Residents’ Perspectives and Intentions to Teach in Outpatient Primary Care Settings

**DOI:** 10.1007/s11606-025-09809-8

**Published:** 2025-08-13

**Authors:** Lawrence Ma, Chad Vercio, Chris Peltier, Caroline R. Paul, Simerjot K. Jassal, Gary Beck Dallaghan, Helen C. Wang

**Affiliations:** 1 University of California, San Diego, San Diego, USA; 2 Loma Linda University, Loma Linda, USA; 3 University of Cincinnati College of Medicine, Cincinnati, USA; 4 NYU Grossman School of Medicine, NYU Langone Health, New York City, USA; 5 University of Texas at Tyler, Tyler, USA; 6 Veterans Affairs Palo Alto, Palo Alto, USA; 7 Stanford University, Stanford, USA; 8 University of Illinois Urbana-Champaign, Champaign, USA; 9 University of Kentucky College of Medicine, Lexington, USA; 10 Department of Pediatrics, Stanford University, 3351 El Camino Real Suite 100, Atherton, CA 94027 USA

**Keywords:** community preceptors, primary care, workforce, qualitative research

## Abstract

**Background:**

Primary care is pivotal to delivering effective healthcare. However, interest in primary care continues to decline; this is compounded by difficulties recruiting and retaining community-based preceptors to train and inspire trainees to enter primary care. Prior research explored the perspectives of community preceptors, but more concerted efforts across primary care specialties need to be directed upstream to understanding residents’ perceptions on becoming future preceptors.

**Objective:**

We aimed to understand the experiences and factors that shape graduating residents’ attitudes about and desire to serve as outpatient preceptors.

**Design:**

This was a qualitative study, using a narrative inquiry approach, based on semi-structured interviews.

**Participants:**

Graduating Internal Medicine, Pediatric, and Internal Medicine-Pediatric residents at a single institution were interviewed.

**Approach:**

We developed an interview guide based on researchers’ own experiences as medical educators and a pilot interview. Applying inductive analysis, we identified common themes that influenced participants’ perspectives on outpatient precepting. With the derived themes, we identified an existing theory that best explained the results.

**Key Results:**

After interviewing 13 residents, four themes were constructed that influence participants’ perspectives on becoming outpatient preceptors. The Theory of Planned Behavior, where one’s intention is informed by one’s behavioral beliefs, normative beliefs, and control beliefs, conceptualized the themes. Witnessed advantages and disadvantages of precepting informed participants’ behavioral beliefs, preparedness to practice medicine and teach informed their control beliefs, physicians’ responsibility to teach contributed to their normative beliefs, and clinical demands were a secondary determinant that influenced all three beliefs.

**Conclusions:**

Using identified themes and the Theory of Planned Behavior, we propose the following recommendations to improve resident outpatient training with the goal of improving long-term community preceptor recruitment: (1) enrich the outpatient learning experience, (2) reinforce the teacher identity, (3) advocate for structural and cultural changes to address current clinical barriers to teaching.

**Supplementary Information:**

The online version contains supplementary material available at 10.1007/s11606-025-09809-8.

## INTRODUCTION

A robust network of primary care physicians is critical for healthcare systems to deliver high-quality care with lower costs.^1,2^ Patients with primary care doctors have lower mortality rates and better self-reported health.^1,2^ By 2036, there is a projected shortage of more than 40,000–58,000 primary care physicians in Internal Medicine, Pediatrics, and Family Medicine.^3,4^ This is compounded by rising numbers of unfilled residency spots in primary care specialties.^4–6^ Medical educators have tried to address this by developing curriculum such as introducing longitudinal primary care programs to increase trainees’ exposure, as this has been shown to influence subsequent career choices.^7,8^ These efforts have been challenging since they require being able to sufficiently recruit and retain community preceptors.^9,10^ The lack of community preceptors willing to teach contributes to the ongoing decline in primary care physicians, resulting in fewer available to teach, inspire, and be models for future generations of trainees.^9,10^

Researchers have sought to address the shortage of community preceptors by understanding the motivational factors and barriers experienced by community physicians who actively teach, have never engaged in teaching, or have stopped teaching.^9–13^ These studies shed light on potential modifiable factors in those who are “settled” into their practices and identities. Although physician identities at this stage may still be malleable,^12,14^ exploring the perspectives of residents, who are earlier on in their medical career, could yield additional actionable insights.

Residents are still developing their professional identity—deciding on how they want to practice, in what setting, and with whom; which means they may be more impressionable.^15^ Additionally, residents are immersed in environments mostly shielded from productivity targets and working in settings that value teaching and require them to be near-peer teachers.^16^ The supervising mandate set by the Accreditation Council for Graduate Medical Education (ACGME) requires residents be given increasing responsibility and opportunities to develop their skill as educators.^16^

Leveraging that teaching momentum after they become practicing physicians is critical as many training programs recruit former graduates to serve as community preceptors. This recruitment process, unfortunately, has become more challenging with fewer graduates willing to precept trainees. Currently, little is known about why residents, who have been near-peer teachers throughout their training, decide not to engage in teaching opportunities upon graduation. In this study, we sought to explore graduating residents’ perspectives on outpatient precepting and the factors that influence their willingness to become outpatient preceptors themselves. By drawing on the experiences of residents from three different primary care residencies (Internal Medicine, Pediatrics, and Internal Medicine-Pediatrics), we hope to gain insight into modifiable factors that can help address the ongoing shortage of outpatient preceptors.

## METHODS

### Study Design

We used a narrative inquiry framework combined with a social constructivism worldview to elicit participants’ stories with an emphasis on their personal experiences interacting with others within the larger medical training environment.^17,18^ The information in these interviews was analyzed with an inductive-analysis approach to generate themes; these themes were subsequently mapped to a preexisting theoretical model.^19,20^

Interview questions were designed based on researchers’ experiences as attendings who precepted learners in the outpatient setting, researchers who investigated the motivational factors of community preceptors, or both. Questions were trialed, modified, and agreed upon by the researchers after a pilot interview. Our one-on-one semi-structured narrative interview consisted of three parts, where the content of the questions was based upon the assumption that experiences and values influence one’s perspectives and intentions. Participants were first asked to describe their experiences as trainees being precepted in the outpatient clinical setting, including both primary care and subspecialty clinical experiences. Then participants were asked how likely they would be to precept medical students and residents based on their future jobs in primary care, subspecialty care, or hospital-based medicine in either academic or non-academic settings. Finally, participants were asked to reflect on the physician’s moral obligation to future generations of trainees. For those who identified as having experiences with precepting junior learners in the outpatient setting, they were asked to reflect on those experiences and the subsequent impacts. Probing questions were included for each part to better explore participants’ stories. The interview guide is available as Digital Appendix S1.

All interviews were conducted by a non-University of California, San Diego (UCSD) affiliated psychologist, with experience in qualitative research, who had no pre-existing or ongoing relationship with the participants.^21^ For the interview, participants used a self-selected pseudonym and were referred to as such by the interviewer. The interviews were conducted and recorded via Zoom (San Jose, CA) between April and June 2023. At the conclusion of each interview, the interviewer asked all participants to complete an anonymous Qualtrics (Provo, UT) survey of demographic data. Interview audio files were transcribed verbatim using Scribie (San Francisco, CA) and reviewed by the Principal Investigator, PI (HW), to ensure they were de-identified prior to the coding process. This study was approved as exempt by the institutional review board (Protocol #801881).

### Setting and Participants

This study was conducted from April 2023 to June 2023 at UCSD, a public academic institution located in San Diego, California. Study participants were all graduating UCSD Internal Medicine, Pediatric, or Internal Medicine-Pediatric residents. We focused on graduating residents to explore how their cumulative clinical experiences shaped their perspectives on outpatient precepting. They offer the richest insights with their diverse exposure to patients, clinical settings (including both primary care and subspecialty care), and different precepting styles. Graduating residents also have more supervisory opportunities including outpatient teaching electives and are best positioned to offer insights regarding their precepting experiences. We intentionally sought participants from Internal Medicine, Pediatrics, or Internal Medicine-Pediatrics because of similarities in how their primary care continuity clinic and subspecialty clinic rotations are structured. In addition, there are ongoing institution-wide challenges in finding community preceptors for medical students and residents in these specialties.

Each of the residency programs is described in more detail below:

- ***Internal Medicine Residency***
- Primary care experiences occurred at Veterans Affairs clinics, academic clinics, and an integrated care model (Kaiser Permanente). Each year, five to seven residents apply to and are selected for the primary care pathway during their first or second year. Only residents in the primary care pathway or the “Resident as Clinician Educator” pathway spend half a day precepting first- and second-year residents at their primary care clinics. Residents had a traditional block schedule with up to 38 weeks of outpatient rotations across 3 years, including two dedicated weeks in their continuity clinic. Residents were required to complete 43 continuity clinic sessions per year. There were 40 graduating residents in 2023; three pursued a career in primary care.
- ***Pediatric Residency***
- All Pediatric residents rotate through three primary care training sites: an academic practice, a federally qualified health center (FQHC), and an integrated care model (Kaiser Permanente). Continuity clinics took place in private practice, FQHC, military-based, integrated care, and academic sites. Each year, two residents are selected for the primary care pathway during their first year. Only residents in the primary care pathway spend 4 weeks precepting first- and second-year residents, pre-clerkship, clerkship, and post-clerkship medical students at two outpatient academic clinic sites. Residents had a traditional block schedule with up to 62 weeks of outpatient rotations across 3 years, including 18 weeks of primary care clinic. Residents were required to complete 36 continuity clinic sessions per year. In 2023, there were 18 graduating categorical pediatric residents; six pursued a career in primary care.
- ***Internal Medicine-Pediatric Residency***
- Primary care experiences were dispersed throughout the clinics listed above. Residents had a traditional block schedule with up to 96 weeks of outpatient rotations, including 12 weeks of primary care clinic. Residents were required to complete at least 36 continuity clinic sessions and see a certain number of adult and pediatric patients per year. There were four graduating residents in 2023; none of the residents pursued a career in primary care.

At the time of this study, only the Veterans Affairs clinic utilized the primary care exception.^22^ This exception allows the supervising attending to not see and evaluate each patient that the resident trainee sees.^22^ For all remaining training sites that did not have the primary care exception, any patient seen by the resident trainee had to be seen and evaluated by the precepting physician.^22^

Residents were recruited via two emails, sent 2 weeks apart, by the Internal Medicine or Pediatric chief residents on behalf of the PI to the entire Internal Medicine, Pediatric, and Internal Medicine-Pediatric residency class of 2023. The recruitment email described the purpose of the study, outlined efforts to maintain the participants’ anonymity (including using a third-party interviewer to generate de-identified transcripts), and provided guarantees that their choices would not have any impact on their clinical rotations or evaluations. Interested participants directly contacted the interviewer to schedule an interview date/time.

### Data Collection and Analysis

Data analysis occurred concurrently with data collection as we reviewed the interview quality. A total of 13 interviews, with an average interview time of 40 min, were conducted. Through concurrent analysis and team discussions, researchers concluded that the responses of the 13 participants were sufficiently diverse to account for varied perspectives and sufficiently comprehensive to allow for the generation of recurrent themes.^23,24^

Researchers utilized Dedoose (Manhattan Beach, CA) to facilitate thematic analysis of the narratives provided by participants.^19^ Researchers were blinded to participant identities, and all researchers analyzed the data. Two transcripts, chosen for maximal length, were inductively coded to construct the initial codebook in whole-group coding meetings. Groups consisting of three researchers each subsequently coded the remaining transcripts, first individually, then together to reconcile their codes.^25^ The PI (HW) participated in every coding session to provide consistency and continuity during the evolution and refinement of our coding schema and compiled every individual’s coding.

Codes were categorized in a meaningful structure, and each investigator constructed overarching themes.^18,19^ In light of the inherent differences of each residency program, we compared codes between specialties and in aggregate, and found no differences. Through group discussion and reconciliation, final themes were developed through an iterative process and were similar across all specialties and in aggregate.^18,19^ Researchers subsequently created a model to illustrate the relationship between the themes. Once the preliminary model was created, researchers performed a literature review on existing theories that best explained our results.^20^ The theory that best aligned with our results was the Theory of Planned Behavior (TPB).

TPB has been used widely across various domains, particularly in public health and business, but it also pertains to clinician behaviors.^26,27^ TPB posits that people are more likely to carry out a behavior if the intent is present.^26,27^ A person’s intent is determined by the interaction between the primary determinants comprised of a person’s behavioral beliefs, normative beliefs, and control beliefs.^26^ Behavioral beliefs refer to the attitudes one has towards a specific behavior.^26^ With behavioral beliefs, an individual is weighing the advantages or disadvantages of carrying out the specific behavior.^26^ Normative beliefs are beliefs brought on by societal pressure or others’ expectations and are influenced by how motivated an individual is to conform to social norms.^26^ Lastly, control beliefs, sometimes referred to as self-efficacy beliefs, describe how easy or difficult it is to carry out the behavior based on how one perceives their own skills and knowledge.^26^ This belief can be influenced by external circumstances such as available resources or opportunities.^26^ Combined, these three components contribute to an individual’s intention, which in turn is a strong predictor of the actual behavior.^26^ In TPB, there are also secondary determinants that act upon each of the primary determinants to then influence an individual’s intention to perform a certain behavior.^26^

### Author Positionality and Reflexivity

Our team consisted of three outpatient primary care pediatricians (CP, CRP, HW), one combined internal medicine and pediatric outpatient primary care physician (LM), one outpatient primary care and hospital medicine pediatrician (CV), one outpatient primary care and hospital medicine internist (SJ) and one doctor of philosophy (GBD). All physicians regularly supervise residents in the outpatient setting. The researchers are also active in medical education through a variety of leadership roles such asrotation site directors (HW, CP, LM), prior clerkship director (CV, CRP), residency program director (SJ), rotation or track director for primary care (CRP, CV), division chief (CV), and assistant and associate medical school deans (GBD and CRP). As mentioned above, several members have also previously published research exploring the perspectives of community preceptors.

Researchers acknowledged that their individual backgrounds, positions, and roles shaped their understanding of the positive and negative experiences residents had in the outpatient setting as many have been in similar situations themselves. The researchers also recognized that their research interests, leadership roles, and personal commitment to advancing medical education influenced the lens through which they interpreted participants’ responses. By using a group analysis process, researchers checked each other on any assumptions or inaccurate interpretations. In these cases, the group would pause to reflect and discuss how individuals’ feelings or experiences may be affecting their interpretation of the data.

## RESULTS

All graduating Internal Medicine, Pediatric, and Internal Medicine-Pediatric residents (N = 62) received an email describing the study. 15 residents expressed interest with 13 completing the interview process. See Table 1 for demographic information of participants.

**Table 1:** Demographics and Career Choices of Participants (*N* = 13)

Demographic	*n* (%)
Age	
26–30 years	9 (69.2%)
31–35 years	3 (23.1%)
36–40 years	1 (7.7%)
Gender	
Female	8 (61.5%)
Residency Program	
Pediatrics	8 (61.5%)
Internal Medicine	4 (30.8%)
Internal Medicine-Pediatrics	1 (7.7%)
Continuity Clinic Characteristics	
Academic clinic	6 (46.2%)
Federally qualified health center	2 (15.4%)
Private practice	4 (30.7%)
Integrated care clinics	1 (7.7%)
Dedicated Outpatient Precepting Clinic Sessions	
None	7 (53.8%)
1	3 (23.1%)
20	3 (23.1%)
Career Choice	
Anticipated career choice immediately after residency*	
Primary Care	10
Fellowship	2
Hospitalist	1
Sports Medicine	1
Urgent Care	2
Setting of immediate anticipated career choice^†^	
Academic	7
Community	9
Underserved	2
Private	2
Not sure	1
After graduation, will precept trainees	
Yes	0 (0%)

^*^Some participants graduated and practiced in both primary care and urgent care settings

^†^Participants selected all settings that they would be practicing in

**Figure 1:**
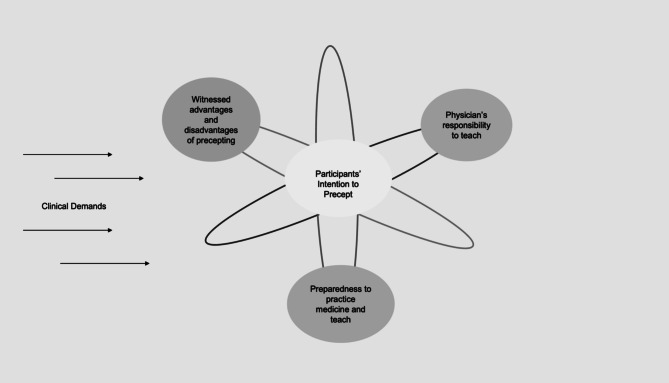
The influences on participants’ intention to precept.

Four themes described the influences on participants’ perspectives of being an outpatient preceptor: (1) witnessed advantages and disadvantages of precepting, (2) preparedness to practice medicine and teach, (3) physician’s responsibility to teach, and (4) clinical demands. Figure 1 conceptualizes the interaction between the themes. To illustrate the interconnectedness of different elements on the overall outcome of a participant’s intention to engage in precepting, consider an atom. The neutrons and protons, which comprise the core of the atom, represent the participant’s intention to precept in the future. The surrounding electric field (clinical demands) and orbiting electrons (represented as: witnessed advantages and disadvantages of precepting, preparedness to practice medicine and teach, and physician’s responsibility to teach) determine what reactions and changes the atom will undergo.

**Figure 2:**
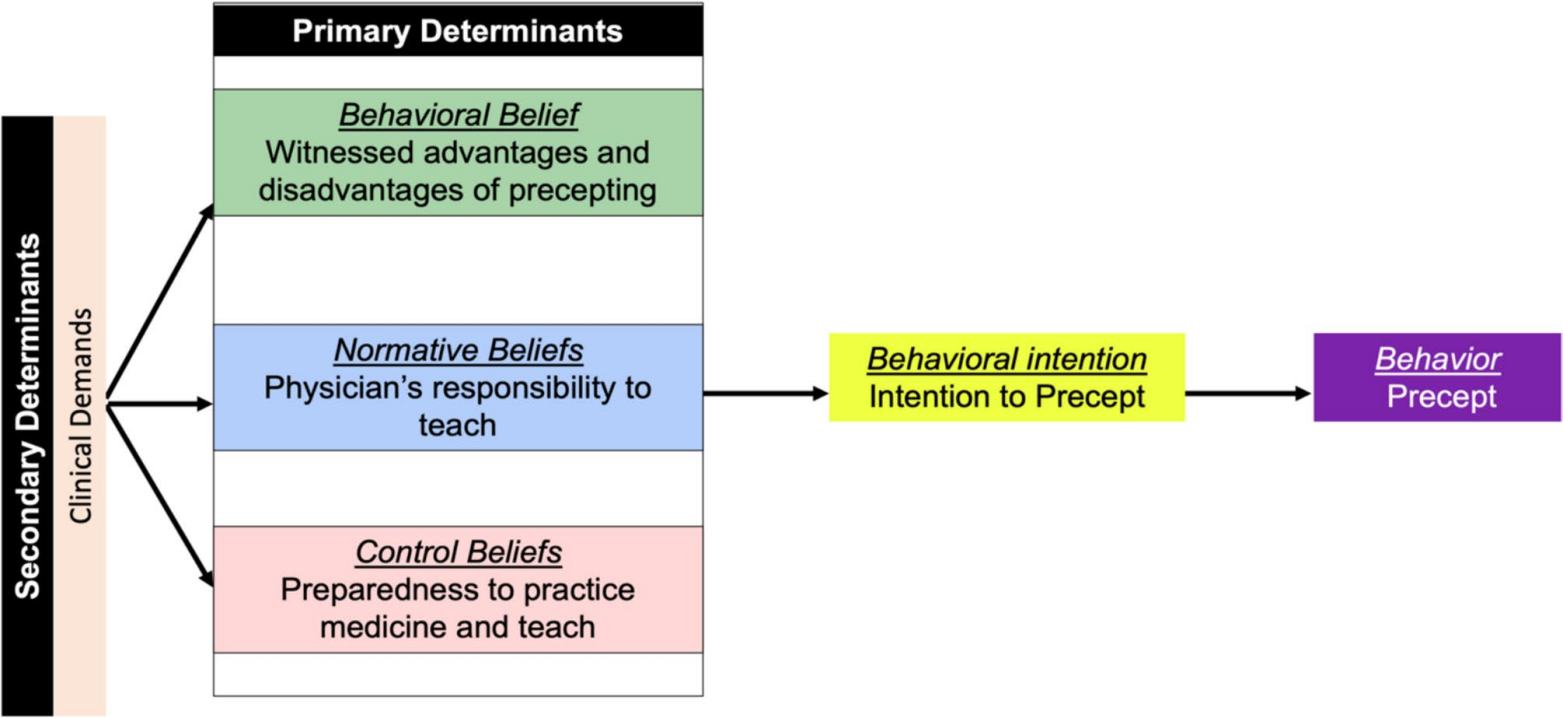
Applying the theory of planned behavior to study themes.

Using the TPB framework,^26^ the specific behavioral outcome of interest was the participant’s intention to precept in the outpatient setting. The participants’ behavioral beliefs were influenced by their perception of the benefits gained or detriments incurred by their preceptors while precepting. Control beliefs were informed by their perceived preparedness to practice medicine and teach. Their normative beliefs were their perceptions about a physician’s responsibility to teach. Clinical demand was a secondary determinant that influenced the participants’ intent to teach by exerting influence on participants’ behavioral, normative, and control beliefs (see Fig. 2).

### Behavioral Belief: Witnessed Advantages and Disadvantages of Precepting

Some participants noted that their preceptors directly benefited from teaching because they had to develop a deeper understanding of medicine and stay more current when actively teaching others.

It’s one thing to learn and practice medicine, but it’s another thing to teach…you need a more nuanced understanding of the material…it keeps you sharp and on your toes. (Participant 8)

Other benefits revolved around being able to provide positive experiences and “give back” to others as their preceptors had done. This first required that their preceptors be “engaged,” “open,” “respectful,” “receptive,” or “excited.” For one participant, the preceptor’s sustained enthusiasm for teaching despite the additional responsibility and time commitment required was clearly appreciated.

We increase the amount of work they have to do…but my preceptor did a really good job of cultivating a positive learning environment by being enthusiastic. (Participant 11)

With these positive learning experiences, they looked forward to being in a position where they could pass on the positive benefits they had gained from their preceptors. In turn, they would feel a sense of “joy,” “fulfillment,” and “reward.”

We’ve all had experiences where we had a great mentor or a great clinical preceptor who taught us to be a good clinician, so I want to be able to share that knowledge. (Participant 12)

Conversely, participants also described disadvantages that came with precepting, including challenges with keeping high patient volume clinics running on time.

… I think it is more challenging to be a resident preceptor because…you don’t know the patients as well…the preceptors precept many residents…the schedules are quite demanding on the resident clinic and…the schedules run very behind. (Participant 12)

The participants also recognized their preceptors had other time demands in addition to clinical care and teaching. They were aware their preceptors were not given “more administrative time to help with some of the other expectations” (Participant 6) which contributed to a palpable sense of “fatigue.”

These challenges resulted in most participants describing precepting in the outpatient setting as “difficult,” “stressful,” “burdensome,” “exhausting,” and “taxing and trying.” One participant spontaneously brought up feelings of burnout.

…many instances when you feel exhausted and kind of burnt out. At the end of a long day when you…wanna get patients through and you’re already behind and then someone tell[s] your medical student ‘go see this patient,’ and they take 30 minutes in the room…you’re even farther behind cause you have to see the patient afterwards. That can get really frustrating. I imagine preceptors who precept residents also feel like that sometimes. (Participant 3)

### Control Belief: Preparedness to Practice Medicine and to Teach

Participants described feeling “terrified,” “nervous,” “anxious,” “fearful,” “hesitant,” and “overwhelmed” about teaching. Many wanted to figure out how to actually “be an [outpatient] attending” before teaching learners.

I’m still trying to figure out how I want to practice. It seems a little overwhelming…I don’t feel that I would be an effective teacher... (Participant 9)

Some worried that if they were to teach, they would be an “imposter” who had little or inaccurate medical knowledge to impart to learners.

I honestly feel like imposter syndrome…Sometimes when I’m teaching people I question myself, I’m like ‘I think that’s right?...What base of knowledge do I have to teach them [learners]?’ (Participant 10)

Multiple participants specifically described lacking confidence in their abilities to teach and evaluate learners in the outpatient setting. Though participants were used to precepting on the inpatient side, they felt the outpatient setting posed challenges, with time management, that they were not used to navigating.

To have all the time to teach in a busy outpatient clinic seems challenging as opposed to an inpatient setting where you have time to do what you need to do. (Participant 1)

This discomfort was also exacerbated by participants feeling they generally spent less time and had limited experience precepting on the outpatient side.

... we don’t have as many opportunities to do outpatient teaching and precepting in a more formal role…in the inpatient setting we often have lots of med students and other learners below us who we’re responsible for. (Participant 7)

For the seven residents who had dedicated opportunities to precept junior learners for either a half-day or for as long as 4 weeks, they all felt that this “reaffirmed” their joy for teaching and improved their outpatient teaching skills.

I’ve been able to practice different skills…it is different than wards teaching, which is where I do the most of my teaching. (Participant 10)

However, the precepting experiences did not change their career decisions. One resident commented that the duration of the rotation was so short that “I don’t know if it’s changed my career goals… or professional identity” (Participant 8).

### Normative Belief: Physician’s Responsibility to Teach

Participants had varying viewpoints about whether teaching was a moral obligation for physicians. Some felt teaching was a moral obligation because they recognized they had learned from someone; therefore, it was their duty to pay it forward. Others described teaching as a responsibility to ensure future generations of physicians were available to continue to care for patients.

... you don’t forget those people who took that extra time with you or really invested in your learning and your maturation as a physician… because we all receive that and it’s so helpful for us, we do have an obligation to the next generation to do that as well. (Participant 10)

However, there were those who felt teaching was an individual choice and teaching trainees was not part of the official job description or responsibility of being a physician. Many felt this choice was primarily determined by where a physician was practicing; teaching should only be expected from those who choose to work in an academic setting.

For people who choose to work in an academic setting surrounded by those learners, you should take that seriously and make that some type of priority. If you’re in private practice or a community and you’re not in that situation, there’s no moral obligation to do formal teaching. (Participant 7)

For others, teaching was for a subset of physicians who were capable of teaching. They echoed the sentiment that not all physicians needed to be teachers. However, the underlying reasoning was because not all physicians were “cut out to be a teacher” or “would be good at teaching.”

I’ve had terrible teachers. People who are great doctors, who are great with patients and are excellent innovators, but they’re not necessarily good teachers. For them…that doesn’t necessarily mean passing on knowledge to the next generation of doctors. (Participant 2)

### Secondary Determinant: Clinical demands

The clinical time pressures that create a tension between clinical care and education/teaching were a secondary determinant to participants’ intention to teach. With learners in the clinic trying to see patients every 15 to 20 min, one participant felt that “education suffers” as “there is not much teaching at all on the outpatient side, because of how busy the schedules are. It’s literally, we’re just trying to survive.” (Participant 6). Several other participants identified lack of protected time and lack of compensation as reasons why they did not intend to teach.

if you don’t get compensated [for teaching], you are going to run behind in your schedule or not have enough time to complete the documentation that is necessary. (Participant 12)

In describing the stressors and disadvantages of teaching while seeing patients, many participants viewed outpatient precepting as impossible and undesirable given “the amount of patients that they’re expected to see and from the inbox requirements as well.” (Participant 6).

## DISCUSSION

**Figure 3:**
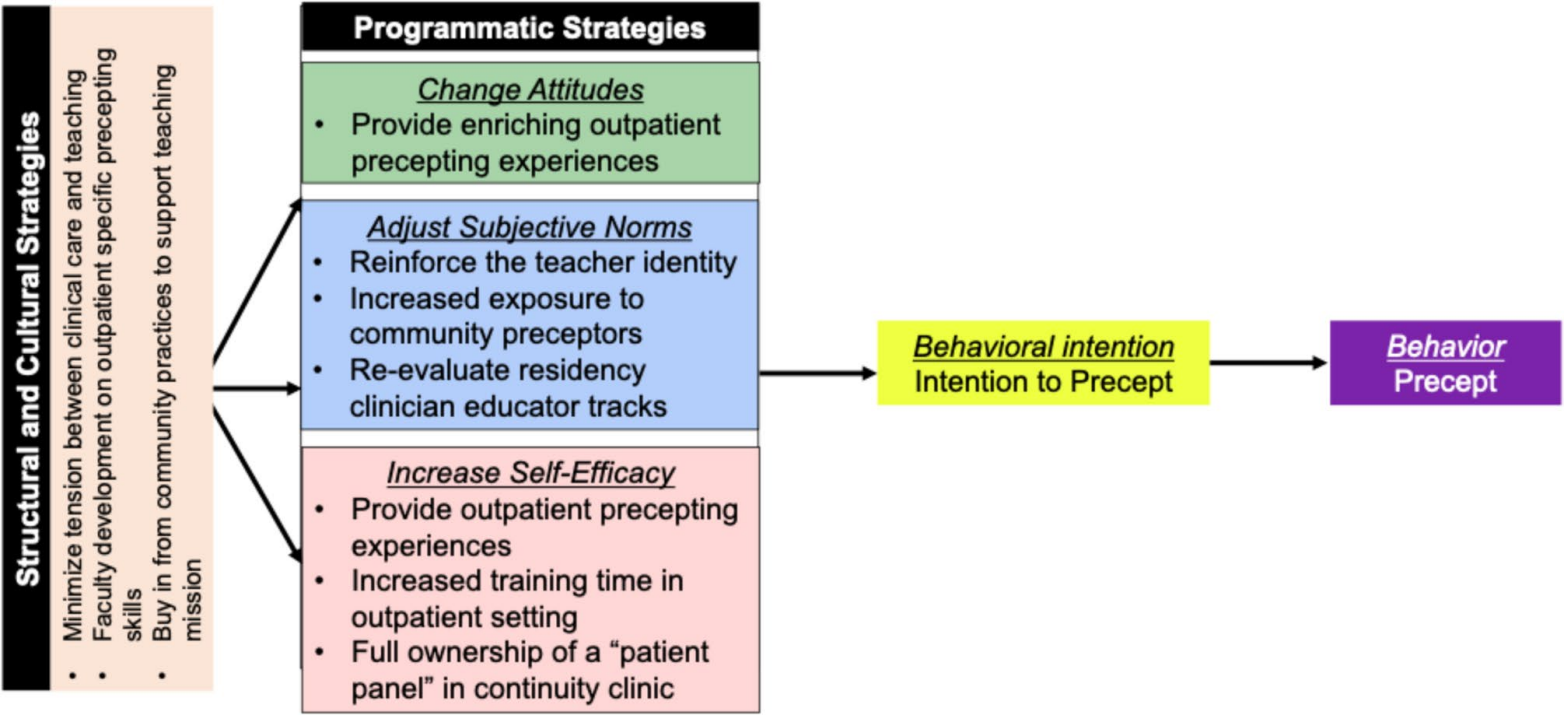
Mapping recommended strategies to the theory of planned behavior.

Our study provides insights on various factors influencing the behavioral, normative, and control beliefs that were shared by graduating Internal Medicine, Pediatric, and combined Internal Medicine-Pediatric residents related to precepting in the outpatient setting. Though some participants described a duty to teach trainees and acknowledged the benefits of precepting, the majority of participants felt unprepared to precept in the outpatient setting. Based on their experiences, we propose training programs across all primary care specialties work together to implement the following recommendations (summarized in Fig. 3) to help influence and promote residents’ intention to precept in the outpatient setting.

### Increase Residents’ Control Beliefs by Building Their Confidence with Outpatient Care

Similar to other reports, our study indicates that graduating residents do not feel confident in their mastery of outpatient care.^28^ Participants cited needing more time to learn how to practice independently as an attending before feeling comfortable precepting learners. These sentiments may originate, in part, from imposter syndrome, but also reflect how residency training programs tend to focus heavily on inpatient settings.^29–31^ Existing outpatient experiences can often feel fragmented and lack the continuity that is so critical for the primary care experience.^32,33^ With new ACGME requirements emphasizing more outpatient experiences, revisiting how outpatient rotations are designed could help future residents feel more confident in their abilities to provide excellent outpatient care.^16^ One way to improve the experience would be ensuring residents spend sufficient continuous time on their outpatient blocks to develop continuity with a panel of patients.^31^ This encourages residents to have more “ownership” over their patients and should boost their comfort and confidence with managing their own patient panels in the future. Learning to manage an electronic health record inbox has also become a critical component of outpatient care and is a skill that needs to be developed with dedicated education on best practices and how to integrate technological innovations including Artificial Intellgience.^34–36^ Any modifications to existing outpatient rotations should consider the impact on preceptors and clinical support staff and avoid adding responsibilities to preceptors without appropriate supportive measures.

### Shape Resident’s Behavioral and Control Beliefs About Outpatient Precepting by Increasing Outpatient Teaching Experiences

As evidenced by other studies and our participants’ experiences, senior residents rarely have opportunities to supervise and educate junior learners in the outpatient setting.^29,30,37^ Residents recognize the need for this subset of supervisory experiences and feel ill-prepared to handle the different challenges that outpatient teaching poses. As described by our participants, these difficulties resulted in the perception that outpatient precepting was not enjoyable and hence not worth pursuing. This is in sharp contrast to the inpatient setting where many residents described feeling more comfortable with teaching.^30^ The inpatient environment has a long-standing and established history of requiring and expecting resident physicians to assume more clinical responsibility and teach junior trainees over time.^29,30,37^ To better prepare residents to precept in the outpatient setting, residency programs need to critically re-evaluate their outpatient curriculum to create the expectation and necessary opportunities for senior residents to supervise junior trainees.^9,30,37^ Creating a safe learning environment through increased protected time for junior trainees, senior residents, and supervising attendings to see patients and complete administrative tasks may lead to a more positive outpatient experience thereby highlighting the rewards of precepting.

In outpatient settings with multiple levels of trainees, this could entail having medical students present their patients to the senior resident.^29^ In the continuity clinic setting, a senior resident could supervise interns and junior residents with an attending present.^29^ These outpatient opportunities would not only reinforce the positive rewards of teaching, but also help residents reinforce their knowledge, build confidence in their precepting abilities, and build their control beliefs. Our results further support that even brief exposures to precepting in the outpatient setting can lead to increased sense of self-efficacy and joy for teaching.^21^

### Reinforce the Teacher Identity to Improve Normative Beliefs

Many medical students, as part of their graduation ceremony in medical school, take some version of the Hippocratic Oath marking their entry into the profession as a medical doctor. The modern abbreviated versions of the Hippocratic Oath often do not include any explicit statement about the responsibility physicians have to educating future generations of physicians. Yet, in the classical translations, this role is one of the core duties of a physician as evident by the statement “to give a share of precepts and oral instruction and all the other learning to my sons [sic] and to the sons of him [sic] who has instructed me and to pupils who have signed the covenant and have taken an oath according to the medical law.”^38^ The very nature and structure of both undergraduate and graduate medical education may actually be undermining who medical trainees perceive should or can be a teacher. From the very beginning of their medical training, students primarily receive their education from academic faculty. The majority of their clerkships as students and rotations as residents are supervised by academic faculty. This creates a norm, as evidenced by multiple participants’ perception, that only academic clinicians are obligated to teach. The popularity and expansion of clinician educator tracks at many institutions may reinforce and inadvertently compound this problem as they divide future graduates into groups that either emphasize teaching or not.^39,40^ This perceived exclusivity runs counter to the very meaning of the word “doctor” which is derived from the Latin word “Docēre” which means to teach.

Additionally, there exists a paradox that all residents are expected to have supervisory roles by the ACGME and therefore are already actively teaching anytime they lead a team with junior trainees.^16^ Yet, most graduating residents do not continue with these teaching roles as attending physicians. Medical schools and residency programs need to re-establish the expectation that being a physician includes the responsibility to teach the next generation. Medical schools need to offer rotations at both academic and community sites across all specialties to ensure students understand that all physicians, and not just a select few, can teach. Residency programs should formalize curriculum on clinical teaching and foster the educator identity for all trainees as opposed to selecting a few residents to develop as clinician educators. Residents also need more consistent opportunities to rotate through community clinic sites.^31,41,42^ By having additional role models in the community, residents can see that being a clinician educator is not a professional identity restricted to academic physicians.

More emphasis also needs to be placed on supporting new attendings to help maintain a community of practice and reinforce the teaching identity.^9,43^ Professional development courses relevant to “real-world” scenarios that partner new attendings with mentors to help them navigate the challenges of managing a diverse clinical practice while teaching will be critical.^9^ Mentorship across primary care specialties could provide additional insights and innovative approaches to address common challenges.

### Influence the Secondary Determinants Through Structural and Cultural Changes

Simply introducing supervisory opportunities for senior residents in the existing outpatient spaces is unlikely to significantly change residents’ perceptions about outpatient precepting; there needs to be co-existing structural and cultural changes.^9^ The bulk of residents’ negative experiences with outpatient precepting in both primary care and subspecialty settings were due to competing priorities between teaching and patient care, inefficient or ineffective preceptors, and a clinical environment perceived to be unsupportive of teaching.^41,44–46^ It will be challenging to convince residents that the rewards of outpatient teaching are worth it if these barriers are not adequately addressed. Training programs can start addressing some of these structural barriers by advocating for more protected time for their preceptors to teach.^10–13^ This would require reductions in the volume of patients seen while teaching and more inbox support so preceptors do not experience significant increases in their non-face-to-face workload while teaching.^9,10,31,41,46,47^ Both of these interventions will result in reduced productivity and carry financial repercussions for the preceptors and their clinics.

Different strategies to address this “financial cost” of teaching could include institutions directly providing monetary compensation, educational tax incentives, or teaching full-time equivalent credits.^12,48–50^ A particular emphasis needs to be placed on providing financial support to primary care preceptors who form the backbone of residency programs’ outpatient continuity experiences and who typically have lower baseline compensation compared to most subspecialists. An indirect financial strategy could include offering new marketing or branding opportunities particularly to community preceptors engaged in teaching.^12,50^ Academic institutions and their training programs also need to place greater emphasis on understanding the unique needs and challenges of their local community physicians to better partner with them in creating new or improved precepting opportunities.^9,12,43,48,50^ Increasing investment in faculty development efforts through peer mentorship programs, accessible practical courses on medical education, and providing Continuing Medical Education or Maintenance of Certificate credits help demonstrate to local preceptors that they are valued members of the medical educator community.^12,48,50^ Lastly, individual specialties and professional organizations should not be addressing these challenges in silos and should partner together to identify innovative solutions and advocate for the necessary institutional changes.^51^

### Strengths and Limitations

These findings should be interpreted with consideration of our study’s strengths and limitations. Strengths include the representation of graduating residents primarily entering primary care who were all in the same graduating year of training from three residency programs. Despite the different numbers of participants from each residency program, the residents shared similar experiences and perspectives on precepting. Incorporating the experiences of trainees beyond Internal Medicine adds to the robustness of the identified themes and demonstrates comparability across different primary care specialties. Another strength of our study includes the diverse experiences of the study researchers, which helps to ensure different perspectives and interpretations are heard.

A key limitation of our study is that it is based on graduating residents from a single academic institution. Additionally, this cohort of participants’ training experiences were heavily impacted by the COVID-19 pandemic, which resulted in significant practice and workflow changes in both the outpatient and inpatient settings. Lastly, only preceptors at the Veteran Affairs clinics supervised residents with the primary care exception. This model of care is widely used at other institutions and could be expected to significantly influence participants’ perceptions about precepting or being precepted. Further expansion of this work should include other academic and community-based residency programs that consistently utilize the primary care exception. Future studies should utilize a mixed methods approach to prioritize themes for more targeted interventions to address the primary care shortage.

## CONCLUSION

The model of medical training has relied on previous generations of physicians teaching the next generation. In the current era of medicine focused on increased productivity and work-life balance, finding preceptors who will consistently take on this role, particularly in primary care, is becoming increasingly challenging. The burdens and the joys of teaching cannot be solely borne by those who choose a career in academics; the increasing number of medical school and residency trainees compared to academic physicians results in an intrinsic supply and demand mismatch. This paper explores the perspectives of graduating residents and describes barriers to taking on the responsibility of precepting and mentoring trainees in the outpatient setting. Their insights should serve as a call to action for medical schools, residency training programs, and professional organizations. Rather than focusing on cultivating a select few to become future educators, we need to create a consistent culture and system that supports and prioritizes a lifelong duty among all physicians to teach medical students and resident trainees and ensure the education of future generations of physicians.

## Supplementary Information

Below is the link to the electronic supplementary material.

Supplementary file1 (DOCX 19 KB)

## Data Availability

The data are not publicly available due to privacy or ethical restrictions. The data that support the findings of this study are available from the corresponding author upon reasonable request.

## References

[1] **O’Mally AS, Rich EC.** Measuring comprehensiveness of primary care: challenge and opportunities. J Gen Intern Med. 2015; 30(Suppl 30: S568-575). 26105670 10.1007/s11606-015-3300-z PMC4512967

[2] **Kroenke K.** The many C’s of primary care. J Gen Intern Ned. 2004; 19(6): 708-709. 10.1111/j.1525-1497.2004.40401.x PMC1492385 15209611

[3] Association of American Colleges. The Complexities of Physician Supply and Demand: Projections from 2021 to 2036. 2024. PP 6–8.

[4] **Bazemore A, Petterson S, McCulloch K.** US Primary Care Workforce Growth: A Decade of Limited Progress, and Projected Needs Through 2040. J Gen Int Med. 2024; 40(2): 339-346. 10.1007/s11606-024-09121-x PMC11802952 39443342

[5] National Resident Matching Program. The Match. Results and Data: 2024 Main Residency Match. 2024. PP 30–55.

[6] American Academy of Family Physicians. 2024 Match. PP1–13.

[7] **Pfarrwaller E, Sommer J, Chung C, et al.** Impact of Interventions to Increase the Proportion of Medical Students Choosing a Primary Care Career: A Systematic Review. J Gen Int Med. 2015; 30(9): 1349-58. 10.1007/s11606-015-3372-9 PMC4539313 26173529

[8] **Obley A, Cooney T.** Fixing the Primary Care Pipeline: The Role of Teaching Health Centers. J Grad Med Educ. 2013; 5(4): 543-544. 24454996 10.4300/JGME-05-04-37 PMC3886445

[9] **Bowen J, Irby D.** Assessing quality and costs of education in the ambulatory setting: a review of the literature. Acad Med. 2022; 77(7): 621-80. 10.1097/00001888-200207000-00006 12114139

[10] **Christner J, Beck Dallaghan G, Briscoe G, et al.** The Community Preceptor Crisis: Recruiting and Retaining Community-Based Faculty to Teach Medical Students - A Shared Perspective From the Alliance for Clinical Education. Teach Learn Med. 2016; 28(3):329-336. 27092852 10.1080/10401334.2016.1152899

[11] **Beck Dallaghan G, Alerte A, Ryan M, et al.** Recruiting and Retaining Community-Based Preceptors: A Multicenter Qualitative Action Study of Pediatric Preceptors. Acad Med. 2017; 92(8): 1168-1174. 28353497 10.1097/ACM.0000000000001667

[12] **Paul C, Vercio C, Tenney-Soiero R, et al.** The Decline in Community Preceptor Teaching Activity: Exploring the Perspectives of Pediatricians Who No Longer Teach Medical Students. Acad Med, 2020; 95(2): 301-309. 31425181 10.1097/ACM.0000000000002947

[13] **Scott I, Sazegar P.** Why community physicians teach students (or not): barriers and opportunities for preceptor recruitment, Med Teach. 2006; 28(6): 5463-565. 10.1080/01421590600627375 17074707

[14] **Monrouxe L, Reese C, Cleland J, et al.** Theoretical perspectives on identity: Research identity in healthcare education. In: Researching Medical Education. 2015; 129–140. Hoboken, NJ: Wiley-Blackwell.

[15] **Barnhoorn P, Nierkens V, Numans M, et al.** General practice residents’ perspectives on their professional identity formation: a qualitative study. BMJ Open. 2022; 12(7):059691. 10.1136/bmjopen-2024-088097 PMC11683983 39732495

[16] ACGME Common Program Requirements (Residency). Accessed February 18, 2025. https://www.acgme.org/globalassets/pfassets/programrequirements/cprresidency_2023.pdf

[17] **Crossley M.** Narrative Psychology, Trauma and the Study of Self/Identity. Theory Pyschol. 2000; 10(4): 527-546.

[18] **Creswell J.** Qualitative INquiry and Research Design: Choosing among Five Traditions. Sage Publications, Inc; 1998:xv 403.

[19] **Braun V, Clarke V.** Using thematic analysis in psychology. Qualitative Research in Psychology. 2006;3(2):77–101.

[20] **Varpio L, Paradis E, Uijtdehaage S, et al.** The Distinctions Between Theory, Theoretical Framework, and Conceptual Framework. Acad Med. 2020; 95(7): 989-994. 31725464 10.1097/ACM.0000000000003075

[21] **Karnieli-Miller O, Strier R, Pessach L.** Power Relations in Qualitative Research. Qualitative Health Research. 2009;19(2):279-289. 10.1177/1049732308329306 19150890 10.1177/1049732308329306

[22] Centers for Medicare & Medicaid Services. Guidelines for Teaching Physicians, Interns & Residents. MLN Booklet. PP12–14.

[23] **Malterud K, Siersma V, Guassora A.** Sample Size in Qualitative Interivew Studies: Guided by Information Power. Qual Health Res. 2016; 26(13):1753-1760. 26613970 10.1177/1049732315617444

[24] **LaDonna K, Artino Jr A, Balmer D.** Beyond the Guise of Saturation: Rigor and Qualitative Interview Data. J Grad Med Educ. 2021; 13(5):607-611. 34721785 10.4300/JGME-D-21-00752.1 PMC8527935

[25] **Cofie N, Braund H, Dalgarno N.** Eight ways to get a grip on intercoder reliability using qualitative-based measures. Can Med Educ J. 2022;13(2):73–6. 35572014 10.36834/cmej.72504 PMC9099179

[26] **Bosnjak M, Ajzen I, Schmidt P.** The Theory of Planned Behavior: Selected Recent Advances and Applications. Eur J Psychol. 2020; 16(3): 352-356. 33680187 10.5964/ejop.v16i3.3107 PMC7909498

[27] **Perkins M, Jensen P, Jaccard J, et al.** Applying Theory-Driven Approaches to Understanding and Modifying Clinicians’ Behavior: What Do We Know? Psychiatr Serv. 2007; 58(3): 342-348. 17325107 10.1176/ps.2007.58.3.342

[28] **Brooks J, Singer S, Rosenthal M, et al.** Feeling inadequate: Residents’ stress and learning at primary care clinics in the United States. Med Teach. 2018; 40(9):920-927. 29228837 10.1080/0142159X.2017.1413236

[29] **Hilburg R, Coyle A.** Resident as Preceptor: An Ambulatory Internal Medicine Curriculum for Third-Year Resident Precepting. MedEd Portal. 2020; 16:11000. 10.15766/mep_2374-8265.11000 PMC7586754 33117888

[30] Ince-Cushman D, Rudkin T, Rosenberg E. Supervised near-peer clinical teaching in the ambulatory clinic: an exploratory study of family medicine residents’ perspectives. Perspect Med Educ. 2015; 4(1):8-13. 25601040 10.1007/s40037-015-0158-z PMC4348229

[31] **Peccoralo L, Callahan K, Stark R, et al.** Primary care training and the evolving healthcare system. Mt Sinai J Med. 2012; 79(4):451-63. 22786734 10.1002/msj.21329

[32] **Gupta R, Davis E, Horton C.** Interval Examination: Building Primary Care Teams in an Urban Academic Teaching Clinic. J Gen Intern Med. Sep 2013; 28(11):1517-1521. 24043568 10.1007/s11606-013-2598-7 PMC3797361

[33] **Butler M, Kim H, Sansone R.** Improved continuity of care in a resident clinic. Clin Teach. 2017; 14(1):45-48. 26748569 10.1111/tct.12489

[34] **Vaa Stelling B, Halvorsen A, Dupras D, et al.** Management of the Electronic Health Record Inbox: Results from a National Survey of Internal Medicine Program Directors. J Grad Med Educ. 2023; 15(6): 711-717. 38045943 10.4300/JGME-D-23-00165.1 PMC10686644

[35] **Robertson S, Robinson M, Reid A.** Electronic Health Record Effects on Work-Life Balance and Burnout Within the I^3^ Population Collaborative. J Grad Med Educ. 2017; 9(4):479-484. 28824762 10.4300/JGME-D-16-00123.1 PMC5559244

[36] **Rajaram A, Hickey Z, Patel N, Newbigging J, Wolfram B.** Training medical students and residents in the use of electronic health records: a systematic review of the literature. J Am Med Inform Assoc. 2020; 27(1):175-180. 31592531 10.1093/jamia/ocz178 PMC7647236

[37] **Chen , Yuan S, Asfaw E, et al.** Near-Peer Supervision in Primary Care: Bringing Teaching Teams From the Wards to the Clinic. J Grad Med Educ. 2023; 15(4): 481–487. 10.4300/JGME-D-22-00830.1 PMC10449350 37637346

[38] Hippocrates. The Genuine Works of Hippocrates. W. Wood; 1886.

[39] **Gracey C, Cantor E, Pessegueiro A.** Clinician-Educator Tracks in Internal Medicine: A National Survey. Am J Med. 2024; 137(10): 1012-1019.e4 38971528 10.1016/j.amjmed.2024.07.001

[40] **Friedman K, Lester J, Young J.** Clinician-Educator Tracks for Trainees in Graduate Medical Education: A Scoping Review. Acad Med. 2019; 94(10):1599-1609. 31169537 10.1097/ACM.0000000000002814

[41] **Keirns C, Bosk C.** Perspective: The Unintended Consequences of Training Residents in Dysfunctional Outpatient Settings. Acad Med. 2008; 83(5):498. 18448907 10.1097/ACM.0b013e31816be3ab

[42] **Arora V, Guardiano S, Donaldson D, et al.** Closing the gap between internal medicine training and practice: Recommendations from recent graduates. Am J Med. 2005; 118(6):680-685. 15922702 10.1016/j.amjmed.2005.03.022

[43] **Gilmer B, Harless C, Gibson L, et al.** Transitioning to rural practice together: a rural fellowship model (in 6 Ps). Rural Remote Health. 2024; 24(4): 8791. 39370370 10.22605/RRH8791

[44] **Bowen J, Salerno S, Chamberlain J, et al.** Changing Habits of Practice. J Gen Intern Med. 2005; 20(12):1181-1187. 16423112 10.1111/j.1525-1497.2005.0248.x PMC1490278

[45] **Baker R, Klein M, Samaan Z, et al.** Exam room presentations and teaching in outpatient pediatrics: effects on visit duration and parent, attending physician, and resident perceptions. Ambul Pediatr. 2007; 7(5):354-9. 17870643 10.1016/j.ambp.2007.05.006

[46] **Schultz K, Kirby J, Delva D, et al.** Medical Students’ and Residents’ preferred site characteristics and preceptor behaviours for learning in the ambulatory setting: a cross-sectional survey. BMC Med Educ. 2004; 4(12). 10.1186/1472-6920-4-12 PMC514563 15298710

[47] **Thomas D, Frambach J, Teunissen P, et al.** Learning in Tension: A Case Study Examining What Internal Medicine Residents Learn in the Ambulatory Care Setting. Perspect Med Educ. 2023; 12(1):41-49. 36908741 10.5334/pme.443 PMC9997111

[48] **Finnell K, Ortiz K, Gowin M, et al.** The Premier Medical Education Model: Improving Preceptor Recruitment in Underserved Areas. Fam Med. 2024; 56(8): 485-491. 39012282 10.22454/FamMed.2024.513346 PMC11412298

[49] **Smith T.** An Update on State Preceptor Tax Incentives: Where Do We Stand? PAEA. 2023. https://paeaonline.org/resources/public-resources/paea-news/an-update-on-state-preceptor-tax-incentives-where-do-we-stand. Accessed February 18, 2025.

[50] **Minor S, Huffman M, Lewis P, et al.** Community Preceptor Perspectives on Recruitment and Retention: The CoPPRR Study. Fam Med. 2019; 51(5):389-398. 31081910 10.22454/FamMed.2019.937544

[51] **Rock D, Grant H.** Why Diverse Teams Are Smarter. Harvard Business Review. 2016.

