# Supplementary material for: Senior Residents’ Perspectives and Intentions to Teach in Outpatient Primary Care Settings
Source: J Gen Intern Med. 2025 Aug 13;40(15):3549–59. doi: 10.1007/s11606-025-09809-8 (PMC12612470; doi:10.1007/s11606-025-09809-8)
Supplement: Supplementary file 1 — Supplementary file1 (DOCX 19 KB) [file 11606_2025_9809_MOESM1_ESM.docx]

Start with the following statements:

1. Hi I am Dr. *** and I will be the one interviewing you today for an IRB exempt study titled “Perspectives on Outpatient Precepting and the “Resident as Outpatient Preceptor” Curricula. You are agreeing to participate in both the 60-minute interview and complete a survey that should only take 2-3 minutes.
2. Thank you for agreeing to participate. I have a list of questions to cover with you today. For all of these questions, please provide specific experiences or interactions you have had. We are interested in the details of your stories. There are no right or wrong answers. Your perspectives and experiences are all valid.
3. Do you have any questions about this study?
4. As a reminder, please keep your audio on at all times.

*Start with question one. You may proceed down the list. Or you may adjust the order of the question to improve conversational flow or to ask for follow up and clarification. Aim to end questions 60 minutes. The goal for all the questions is to solicit specific stories and experiences. Please prompt them if they do not provide stories or experiences.*

**Interview Questions:**

1. What does precepting in the outpatient setting look like to you?
   1. What have you perceived to be good/effective clinical teaching? *Please give examples.*
   2. What have you perceived to be less effective clinical teaching? *Please give examples.*
2. What feelings come to mind when you think about precepting medical students or residents in the outpatient setting?  *(Give prompts if they have nothing: excited, apprehensive, neutral) etc.*
   1. Why do you feel this way? Give examples.
3. On a scale from 1-5 (1 being not at all, 5 being absolutely yes), how likely do you see yourself as a preceptor for medical students in the future?
   1. Why did you choose that number?
   2. [Skip if answered with “1”] Why did you not choose a lower number?
   3. What experiences have contributed to you choosing the number [insert number]?
   4. [Skip if answered with “5”] What would have to happen for you to choose a higher number? Please be specific. *(You may prompt them if they say, ‘if I was given time to teach’, with questions about what would your day to day workflow have to look like?)*
4. On a scale from 1-5 (1 being not at all, 5 being absolutely yes), how likely do you see yourself as a preceptor for residents in the future?
   1. Why did you choose that number?
   2. [Skip if answered with “1”] Why did you not choose a lower number?
   3. What experiences have contributed to you choosing the number [insert number]?
   4. [Skip if answered with “5”] What would have to happen for you to choose a higher number? Please be specific. *(You may prompt them if they say, ‘if I was given time to teach’, with questions about what would your day to day workflow have to look like?)*
5. Are there moral obligations (or duties) physicians have to the next generations of medical students and residents?
   1. If answers YES:

What are they?

Why do you feel this way? *Please provide a rationale for your answer.*

*If they don’t cover teaching at all as an obligation then please ask:” how does teaching fit into all of this?”*

- 1. If answers NO:

Why do you feel this way? *Please provide a rationale for your answer.*

**ONLY For those who have precepted in the outpatient setting:**

1. How has this rotation, where you precepted junior learners in the outpatient clinical setting, affected
   1. Your career goals
   2. Your professional identity, in other words, the kind of doctor you see yourself as
   3. Your outpatient clinical teaching skills
   4. Your likelihood to be a community preceptor in the future when you graduate residency or fellowship? Why is it [increased/decreased]?

*At the end of the session time (approximately 60 minutes), please post the Qualtrics Link into the chatbox. Link is* **https://ucsd.co1.qualtrics.com/jfe/form/SV_0JuK0oRkmk4ufjg**

Please answer this short Qualtrics survey that should take only 2-3 minutes. When you complete the survey, you will be asked to provide your email. Your e-mail will not be linked to any of your interview or survey data. You may leave this interview once you have completed the Qualtrics survey.

**Qualtrics survey after interviews:**

Q1. What age range are you in?

21-24

25-30

31-34

35-40

41-44

>45

Q2. With which gender do you most identify with at this time?

Female

Male

Non-binary/gender fluid

Transgender female

Transgender male

In my own word: __

Prefer not to answer

Q3. What is your anticipated career choice immediately after residency (for example: fellowship, hospitalist, primary care, urgent care, etc)?

________

Q4. What setting will your immediate anticipated career choice be in (select all that apply)?

Academic

Community

Underserved

Private

Not sure

None of the above: ____

Q5. LIKERTS: 6-point Likert scale (1 = s*trongly disagree*, 2 = *disagree*, 3 = *somewhat disagree*, 4 = *somewhat agree*, 5 = *agree*, 6 = *strongly agree*

- I am comfortable precepting in the outpatient setting.
- I have the skills to teach in the outpatient setting.
- I feel prepared to supervise physicians in training in the outpatient setting.
- I feel prepared to supervise medical students in the outpatient setting.
- The current residency structure provides me with opportunities to teach in the outpatient setting.
- Outpatient precepting would be a valuable part of the residency curriculum.
- It is my moral obligation as a physician to teach the next generation of learners.

Those who completed the rotation, additional questions:

Q6. LIKERTS: 6-point Likert scale (1 = s*trongly disagree*, 2 = *disagree*, 3 = *somewhat disagree*, 4 = *somewhat agree*, 5 = *agree*, 6 = *strongly agree*

- I enjoyed precepting in the outpatient clinic setting.
- Precepting in the outpatient clinic was a valuable experience.
